# Bone Material Properties of Human Phalanges Using Vickers Indentation

**DOI:** 10.1111/os.12455

**Published:** 2019-04-30

**Authors:** Bing Yin, Jia‐liang Guo, Jian‐zhao Wang, Sheng Li, Ya‐ke Liu, Ying‐ze Zhang

**Affiliations:** ^1^ Department of Orthopaedic Surgery The Third Hospital of Hebei Medical University Shijiazhuang Hebei China; ^2^ Key Laboratory of Biomechanics of Hebei Province Shijiazhuang Hebei China; ^3^ Department of Orthopaedic Surgery Affiliated Hospital of Nantong University Nantong Jiangsu China; ^4^ Chinese Academy of Engineering Beijing China

**Keywords:** Bone hardness, Vickers hardness, Hardness distribution, Biomechanics

## Abstract

**Objective:**

To investigate the microhardness distribution throughout the human hand phalanges using the Vickers method, which can be used to directly evaluate the bone mechanical properties at tissue level and provide an alternative means to investigate bone quality.

**Methods:**

The phalanges bones involved in this study were collected from three healthy donors; fresh‐frozen right limbs were used. The phalanges bones were dissected and cut into 3‐mm thick slices perpendicular to the long axis in the phalanges base, the phalanges shaft, and the phalanges head with a low‐speed saw and then the slices were polished with sandpaper. A microindenter fitted with a Vickers indenter point was used to measure the Vickers hardness in the plantar, dorsal, medial, and lateral sites of cortical bone in metatarsal shaft and trabecular bone in the metatarsal base and head. The indentation load and dwell time was set to 50 g and 12 s for both the cortical and cancellous tissues in this study. For each site or region, five valid values were recorded and averaged as the Vickers hardness for the site or region.

**Results:**

In total, 96 bone slices were harvested from the base, shaft, and head of the 15 phalanges and 1920 indentations were performed. In general, the Vickers hardness in phalanges was 34.11 ± 7.95 HV. For the 5 phalanges, the 3rd phalanx showed the highest hardness (36.74 ± 7.10 HV), closely followed by the 1st (36.46 ± 5.96 HV) and 2nd (35.28 ± 6.52 HV) phalanx. The hardness in the 4th (31.90 ± 9.15 HV) and 5th (31.19 ± 8.22 HV) phalanx were significantly lower than in the other 3 phalanges. The hardness in the phalanx shaft (38.52 ± 6.67 HV) was significantly higher than that in both the base (30.73 ± 7.46 HV) and head (30.64 ± 6.81 HV) of the phalanx (*F* = 300.7, *P* = 0.000); no statistic difference existed between the base and head of the phalanx (*P* = 0.996). The Vickers hardness in the proximal, middle, and distal phalanx showed statistical difference in Vickers hardness (*F* = 19.278, *P* = 0.000). The proximal phalanx showed higher Vickers hardness than the middle phalanx in the 2nd to 5th phalanges (*P* = 0.002).

**Conclusion:**

This study reported on the Vickers hardness distribution of the human phalanges bone and provides the theoretical basis of differences in hardness, which will benefit the placement of plates and screws in orthopaedic surgery and contribute to the research on ideal artificial bones and 3D‐printed orthopaedic implants with inner gradient distribution of hardness.

## Introductions

Bone quality is an important consideration for orthopaedic surgeons in the clinic, as bone quality directly impacts the risk of fracture, the treatment methods of fracture, as well as rehabilitation and prognosis. Bone quality is affected by geometry, microarchitecture, cortical porosity, degree of mineralization, and micro‐damage^1^, ^2^. In clinic, dual‐energy X‐ray absorptiometry (DEXA) and high‐resolution quantitative computed tomography (HR‐qCT) are typically used in the investigation of bone quality^1^, ^2^. Clinical and laboratory research report that in addition to bone mineral density (BMD), the mechanical properties of bone tissue are expected to play a critical role in bone strength and fracture risk^3^, ^4^, ^5^. However, those experimental studies which were performed at the biopsy and organ levels have demonstrated that BMD and microstructure are not enough to explain the variation in bone strength^6^, ^7^.

Bone is an anisotropic and inhomogeneous composite at the micro and nano scale, and the degree of anisotropy can vary and has been simply described as transversely isotropic or orthotropic^8^. The constituents at the nano and micro scales are hard calcium mineral crystals and a softer collagen matrix. Therefore, experimental studies^7^ have found that the bone mechanical properties at the micro scale vary in different bones, sites, and regions. From the first half of the 20th century to recent years, bone hardness has provided an alternative option in the evaluation of bone mechanical properties^9^, ^10^, ^11^, ^12^. In past years, indentation at the micro scale was often used and regarded as an effective tool in the detection of homogeneous material properties, including hardness, elastic modulus, and fracture toughness^8^, but could not distinguish the variation of bone properties within different bone sites and regions.

Therefore, there is a necessity for better detection and understanding of bone mechanical properties at the tissue level^3^. This is not only important for more effective assessment of fracture risk but, eventually, also for treatment instruction. Moreover, this research could benefit the assessment of the effect of drugs on bone strength without the need for large and expensive prospective fracture trials. Micro‐indentation, a unique method, can directly measure the bone hardness at tissue level and accurately evaluate the bone mechanical property, and even indicate some diseases such as osteoporosis and metabolic disorders^9^, ^10^, ^13^, ^14^, ^15^. Micro‐indentations could be applied in both cortical and trabecular bones to detect the mechanical properties at the tissue level^16^, ^17^, ^18^. This technique was performed on a single bone slice using a Vickers or Knoop indenter with appropriate loads^17^. The Vickers test examines tissues covering osteons and interstitial tissues using a pyramid shaped indenter made of diamond^18^. The current 3D‐printing technique can only restore the bone shape and produce artificial bone structure with homogeneous structure, which can be regarded as the traditional 3D‐printing implants. Those orthopaedic implants and artificial bones were produced by materials with higher hardness and elasticity modulus, and do not match the inhomogeneous distribution of bone hardness^19^. Therefore, the Vickers indentation technique could also be used in the design of 3D printing implants, artificial bone, joint prosthesis, and internal fixators for orthopaedic treatment.

Previous studies on bone Vickers hardness have provided some data and independent variables in conducting the Vickers measurement, including applied mass, dwell time, drying time, time between indentation and measurement, and distance between the center of an indentation and the edge of other indentations and pores^8^. However, to the authors’ knowledge, none of those studies had revealed the hardness of human phalanges. The movement of the phalanges contributes greatly to the function of the hand and phalanges fractures comprise a large percentage of human traumatic fractures^20^. Investigation of human phalanges hardness could benefit the understanding of the biomechanical properties of phalanges bone at tissue level. The results could be helpful for analysis of the relationship between hardness and stress transmission. Knowing the hardness distribution associated with the epidemiology of the phalanges fractures may benefit fracture prediction and reduce fracture risk. This article, drawing on the research of our predecessors, investigated the Vickers hardness distribution of human phalanges.

## Materials and Methods

### *Sample Preparation*

**Figure 1:**
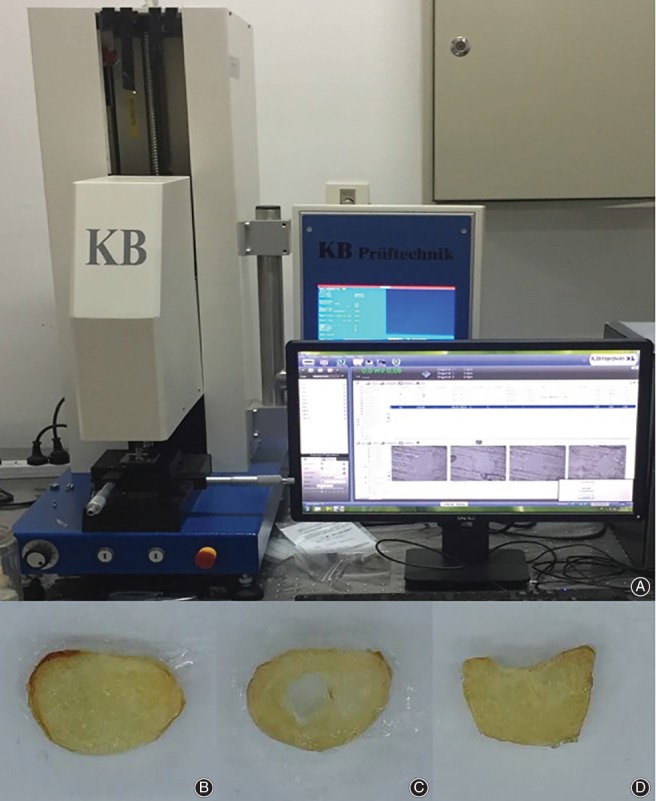
(A) The micro‐indenter used in the study (Model KB5BVZ‐Video, Germany). (B) Bone slice of the phalanges base. (C) Bone slice of the phalanges shaft. (D) Bone slice of the phalanges head.

The phalange bones in this study were taken from right limbs of three donors, whose ages were 62 (male, Donor A), 45 (female, Donor B), and 58 years (male, Donor C). Previous studies found no statistically significant difference in hardness between bilateral bones^11^, ^21^, ^22^. The diagnosis of osteoporosis, metabolic bone diseases, bone tumors, and hormonal imbalance had been excluded. The phalange bones were dissected from the fresh‐frozen specimens. After the ligaments and soft tissue were stripped off, the bones were cut into 3‐mm‐thick slices perpendicular to the long axis using a low‐speed‐diamond saw (Fig. 1B–D). These slices were stuck onto the glass sheets with epoxy resin and polished with increasingly fine sandpaper for a smooth surface. When the procedures were finished, the specimens were wrapped with plastic film to avoid dehydration and stored at −20 °C. This study was approved by the Ethics Committee of the Third Hospital of Hebei Medical University and all aspects of the study comply with the Declaration of Helsinki.

### *Vickers Indentation Performance and Data Collection*

**Figure 2:**
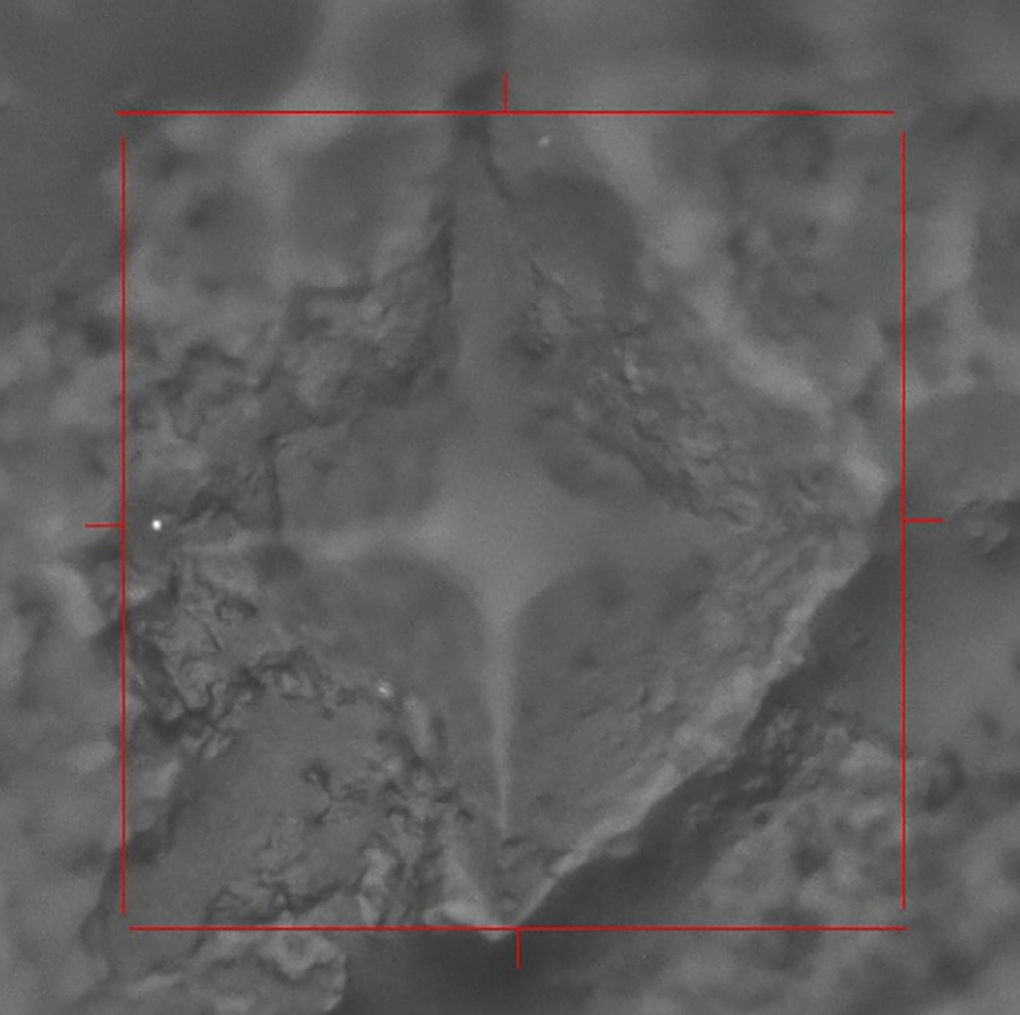
The image of Vickers indentation of human phalanges.

Before testing, the specimens were kept at room temperature for 15 minutes and then blotting paper was used to soak up the excess tissue fluid from the tissue surface. A micro‐indenter (Model KB5BVZ‐Video, Germany) fitted with a Vickers indenter point was used in the measurement (Fig. 1A). Based on previous works^21^, ^23^, the indentation load and dwell time was set to 50 g and 12 s for both the cortical and trabecula regions. The lengths of diagonals were measured with the assistance of the reflected light microscopy and the Vickers hardness value (HV) was calculated. A minimum distance of 2.5 diagonals from the border was always used to avoid boundary effects and a minimum distance of 2.5 diagonals between indentations was established to avoid overlapping of deformation from one indentation to another^24^. The distance from the indentation center to the Haversian canal edge should be at least 60 μm to assure the absence of border effects^8^, ^21^. Asymmetric indentations with differences of more than 5% between diagonals were discarded, and the indentation was repeated^11^, ^13^, ^25^. For each site or region, five valid values were recorded and averaged as the Vickers hardness for this site or region. Fig. 2 shows the image of Vickers indentation of human phalanges.

### *Statistical Analysis*

Statistical analysis was performed with the SPSS statistical package (version 13.0; SPSS, Chicago, IL) with an alpha risk level defined at 5%. The data, which follow the normality distribution, are expressed as mean ± standard deviation (mean ± SD) and were compared using one‐way analysis of variance (ANOVA) followed by an LSD or Dunnett's T3 post‐hoc test. Significance was determined by *P* < 0.05.

## Results

### *General Hardness Distribution*

In three donors, 96 bone slices were harvested from the base, shaft, and head of the 15 phalanges. In total, 1920 indentations were performed at the palmar, dorsal, medial, and lateral regions in each slices. Fig. 1 shows the Vickers indentation of human phalanges. In general, the Vickers hardness in phalanges was 34.11 ± 7.95 HV. Table 1 presents the exact figures for the phalanges hardness. For the 5 phalanges, the 3rd phalanx shows the greatest hardness (36.74 ± 7.10 HV), closely followed by the 1st (36.46 ± 5.96 HV) and 2nd (35.28 ± 6.52 HV) phalanx. The hardness in the 4th (31.90 ± 9.15 HV) and 5th (31.19 ± 8.22 HV) phalanx were significantly lower than those of the other three phalanges.

**Table 1:** Vickers hardness value (HV) distribution in phalanges (mean±SD)

Phalanx	Phalanx base	Phalanx base	Phalanx shaft	Phalanx head	Mean ± SD	Mean ± SD
1st phalanx	Proximal phalanx	36.01 ± 4.71 (60)	41.05 ± 4.03 (60)	35.79 ± 5.40 (60)	37.62 ± 5.31 (180)	36.46 ± 5.96 (240)
	Distal phalanx		32.98 ± 6.46 (60)		32.98 ± 6.46 (60)	
2nd phalanx	Proximal phalanx	31.54 ± 4.10 (60)	36.17 ± 4.93 (60)	29.23 ± 5.55 (60)	32.31 ± 5.66 (180)	35.28 ± 6.52 (420)
	Middle phalanx	33.33 ± 6.08 (60)	43.72 ± 3.74 (60)	33.04 ± 3.49 (60)	36.70 ± 6.75 (180)	
	Distal phalanx		39.91 ± 3.37 (60)		39.91 ± 3.37 (60)	
3rd phalanx	Proximal phalanx	36.29 ± 8.01 (60)	42.13 ± 5.93 (60)	36.05 ± 7.23 (60)	38.16 ± 7.61(180)	36.74 ± 7.10 (420)
	Middle phalanx	31.97 ± 6.34 (60)	42.20 ± 4.11 (60)	33.56 ± 5.17 (60)	35.91 ± 6.92 (180)	
	Distal phalanx		34.97 ± 5.08 (60)		34.97 ± 5.08 (60)	
4th phalanx	Proximal phalanx	28.23 ± 6.89 (60)	40.68 ± 8.49 (60)	26.41 ± 5.78 (60)	31.78 ± 9.53 (180)	31.90 ± 9.15 (420)
	Middle phalanx	26.38 ± 7.25 (60)	38.09 ± 3.94 (60)	25.26 ± 8.42 (60)	29.91 ± 8.93 (180)	
	Distal phalanx		38.26 ± 4.86 (60)		38.26 ± 4.86 (60)	
5th phalanx	Proximal phalanx	26.94 ± 6.04 (60)	40.45 ± 9.82 (60)	28.29 ± 4.32 (60)	31.89 ± 9.33 (180)	31.19 ± 8.22 (420)
	Middle phalanx	25.89 ± 7.63 (60)	33.61 ± 5.07 (60)	28.17 ± 4.06 (60)	29.22 ± 6.61 (180)	
	Distal phalanx		35.01 ± 7.45 (60)		35.01 ± 7.45 (60)	
Total		30.73 ± 7.46	38.52 ± 6.67	30.64 ± 6.81	34.11 ± 7.95	

### *Hardness of Different Bone Sites*

The hardness in the phalanx shaft (38.52 ± 6.67 HV) was significantly higher than that in both the base (30.73 ± 7.46 HV) and head (30.64 ± 6.81 HV) of the phalanx (*F* = 300.7, *P* = 0.000); no statistic difference existed between the base and the head of the phalanx (*P* = 0.996). The Vickers hardness in the proximal, middle, and distal phalanx showed statistical difference in Vickers hardness (*F* = 19.278, *P* = 0.000). The proximal phalanx showed higher Vickers hardness than the middle phalanx in the 2nd to 5th phalanges (*P* = 0.002). The hardness in the palmar (33.93 ± 8.02 HV), dorsal (33.69 ± 8.16 HV), medial (34.31 ± 8.24 HV), and lateral sites (34.52 ± 7.35 HV) showed statistically consistent results (*F* = 1.048, *P* = 0.370) (Table 2).

**Table 2:** Vickers hardness value (HV) distribution in palmar, dorsal, medial, and lateral site of phalanges (mean±SD)

Locations	1st phalanx	2nd phalanx	3rd phalanx	4th phalanx	5th phalanx	Total
Palmar site	33.90 ± 5.76 (60)	34.97 ± 6.38 (105)	36.71 ± 6.05 (105)	31.34 ± 10.63 (105)	32.71 ± 8.34 (105)	33.93 ± 8.02 (480)
Dorsal site	39.69 ± 5.24 (60)	35.18 ± 6.42 (105)	33.68 ± 7.75 (105)	31.72 ± 9.28 (105)	30.75 ± 8.28 (105)	33.69 ± 8.16 (480)
Medial site	36.85 ± 5.13 (60)	37.24 ± 6.30 (105)	38.41 ± 7.36 (105)	30.58 ± 9.78 (105)	29.55 ± 6.49 (105)	34.31 ± 8.24 (480)
Lateral site	35.39 ± 6.19 (60)	33.70 ± 6.57 (105)	38.15 ± 6.19 (105)	33.96 ± 6.02 (105)	31.76 ± 9.31 (105)	34.52 ± 7.35 (480)
Total	36.46 ± 5.96 (240)	35.28 ± 6.52 (420)	36.74 ± 7.10 (420)	31.90 ± 9.15 (420)	31.19 ± 8.22 (420)	34.11 ± 7.95 (480)

## Discussion

### *Aims of the Study*

In this study, we systematically measured the Vickers hardness of the phalanx bones as part of the Human Bone Hardness Research Projects. Vickers hardness, as a direct method for evaluation of mechanical properties^12^, ^26^, provided accurate data on different bones, sites, and regions^15^. The results are helpful for understanding the biomechanical properties of bone at tissue level as well as the relationship between the hardness with load bearing and stress transmission. In our study, 96 bone sites and regions were involved and 1920 indentations were performed. To our knowledge, this is the first systematic research on the Vickers hardness of human phalanges.

### *General Hardness Distribution of Human Phalanges*

In our study, the phalanx bone showed the same distribution trend with the long bone hardness. The hardness of the phalanx shaft was significantly higher than that of the phalanx base and head. This result was in line with Weaver and Ohman's report^11^, ^14^. Ohman *et al.* 11 measured 6 long bones and found that the cortical bone tissue was harder (+18%) than the metaphysis. In our study, the phalanx shaft was much harder (+14%–54%) than the phalanx base and head. A common characteristic of these bones is that the bone shaft is mainly cortical bone, whereas the proximal and distal sites are mainly trabecular bone. Hardness of trabecular bone was reported to be lower (10%–15%) than that of the interstitial bone of the adjacent cortex^9^, ^13^.

Bone hardness can be regarded as a marker for load transmission and indicates the load bearing at different bones and sites^19^. In this study, the 3rd phalanx showed the highest hardness value, followed by the 1st and 2nd phalanx. The hardness in the 4th and 5th phalanx was significantly lower than in the other three phalanges. In Marion's grip‐force testing, the 1st, 2nd, and 3rd phalanx provide 60% of total grip force, whereas the 4th and 5th phalanx only provide 18%^27^. Previous research concluded that bone shape and structure molding can be affected by loading transmission^19^.

### *Prospects and Limitations*

This study reported on the hardness distribution of human phalanx bone, and how the differences in bone hardness between different sites and regions would benefit the choice of position of plates and screws, the density and number of screws placed, and the direction of screw placement in the internal fixation of fractures. The choice and placement of the plate and screw should take into consideration the differences in local bone hardness. In an area with greater hardness, fewer screws with fine threads can obtain enough stabilization and maintain the local blood supply. In contrast, in an area with low hardness, coarse thread screws combined with bicortical fixation are more liable to provide proper stabilization. Insufficient screws or unreasonable direction can result in screw and plate loosening, bone non‐union, or other complications after internal fixation of fracture fixation. Placement of screws in regions with a higher trabecular BMD is regarded to prevent implant loosening and may improve patient outcomes^28^. Therefore, orthopaedic surgeons should fully recognize the distribution of human bone hardness preoperatively, so as to achieve more satisfactory efficacy. The results would also contribute to the research on ideal artificial bone and the design of 3D‐printed orthopaedic implants. The 3D‐printing technique could restore the bone shape but produce artificial bone with a homogeneous structure, which does not accord with gradient distribution of bone hardness physiologically^19^. Therefore, our results provide a theoretical basis and valuable data that is expected to be effective in further study regarding the production of an ideal framework for new bone scaffold designs of advanced bone substitute applications.

However, our current study only focuses on hardness distribution of healthy bone tissue. Further study will be needed to investigate the variation of hardness between physiological and pathological conditions, younger and older people, and males and females.
